# Profile of MicroRNAs following Rat Sciatic Nerve Injury by Deep Sequencing: Implication for Mechanisms of Nerve Regeneration

**DOI:** 10.1371/journal.pone.0024612

**Published:** 2011-09-13

**Authors:** Bin Yu, Songlin Zhou, Yongjun Wang, Guohui Ding, Fei Ding, Xiaosong Gu

**Affiliations:** 1 Jiangsu Key Laboratory of Neuroregeneration, Nantong University, Nantong, China; 2 Key Lab of Systems Biology, Shanghai Institutes for Biological Sciences, Chinese Academy of Sciences, Shanghai, China; New York State Institute for Basic Research, United States of America

## Abstract

Unlike the central nervous system, peripheral nerves can regenerate when damaged. MicroRNA (miRNA) is a novel class of small, non-coding RNA that regulates gene expression at the post-transcriptional level. Here, we report regular alterations of miRNA expression following rat sciatic nerve injury using deep sequencing. We harvested dorsal root ganglia tissues and the proximal stumps of the nerve, and identified 201 and 225 known miRNAs with significant expression variance at five time points in these tissues after sciatic nerve transaction, respectively. Subsequently, hierarchical clustering, miRNA expression pattern and co-expression network were performed. We screened out specific miRNAs and further obtained the intersection genes through target analysis software (Targetscan and miRanda). Moreover, GO and KEGG enrichment analyses of these intersection genes were performed. The bioinformatics analysis indicated that the potential targets for these miRNAs were involved in nerve regeneration, including neurogenesis, neuron differentiation, vesicle-mediated transport, homophilic cell adhesion and negative regulation of programmed cell death that were known to play important roles in regulating nerve repair. Finally, we combined differentially expressed mRNA with the predicted targets for selecting inverse miRNA-target pairs. Our results show that the abnormal expression of miRNA may contribute to illustrate the molecular mechanisms of nerve regeneration and that miRNAs are potential targets for therapeutic interventions and may enhance intrinsic regenerative ability.

## Introduction

The peripheral nervous system (PNS), differing from the central nervous system (CNS), has the intrinsic capacity to regenerate. Previous studies have demonstrated that severed peripheral nerves are able to re-grow and re-connect to their targets, even if their previous functions were seriously compromised [1]. As we know, nerve regeneration is a complex biological phenomenon incorporating multiple cells, growth factors and extracellular matrices [2], [3]. In order to achieve successful nerve repair, neuronal loss has to be prevented, axons have to re-grow and arrive at their correct target cells [4]. There is, of particular interest, a growing consensus that the distinct ability of peripheral nerves to re-grow to their targets hinges on the regenerative properties of its glia, the Schwann cells (SCs). Following adult peripheral nerve axotomy, dedifferentiated SCs can replenish lost or damaged tissue by proliferating, and produce various trophic factors and adhesion molecules to facilitate axon outgrowth [5]. In addition, two-way communication between neuron and SC is essential for axonal conduction in axon regeneration. SCs can regulate synapse formation, can control synaptic strength, and may participate in information processing by coordinating activity among sets of neurons. Conversely, neural impulse activity regulates a wide range of SC activities, including proliferation, differentiation, and myelination [6]. However, the widely elucidatory molecular mechanisms that are responsible for PNS injury and the subsequent restoration of nerve remain largely unclear.

MiRNAs are endogenous, non-coding 21- to 23-nucleotide small RNA molecules that regulate gene expression by binding to the 3′-untranslated region of target mRNAs, leading to their translational inhibition or degradation [7]. Many studies have indicated miRNAs are attractive candidates as upstream regulators, because miRNAs can post-transcriptionally regulate the entire set of genes [8]. The importance of miRNA in neural development and neurodegeneration is starting to be recognized [9], [10], but their roles in nerve injury and repair currently remains largely unknown. It was reported that miRNA expression profiles were significantly altered in the spinal cord injury model of adult rats [11], [12]. Recently, miRNA-144, 145, and 214 are identified to be down-regulated in primary neurons responding to sciatic nerve transection, and miR-145 inhibited neurite growth of dorsal root ganglia (DRG) neuron through Slit-Robo-srGAP signaling pathway [13]. In particular, through microarray we also found that abnormal expression of miRNA in DRG may contribute to illustrate the molecular mechanisms of nerve regeneration during the early phase after sciatic nerve transection [14].

Since deep sequencing overcomes the technical drawbacks of microarray and traditional small RNA library sequencing, and has several additional advantages, such as high resolution, high-throughput, high-accuracy and reduced complexity of experimental procedures, it has dramatically changed the speed of all aspects of sequencing in a rapid and cost-effective fashion. This innovation can permit unbiased, quantitative and in-depth investigation of the small RNA transcriptome, and has been widely employed to reveal the expression profiles of miRNA in different species and understand the role of miRNA in fundamental processes [15]–[17].

**Figure 1:**
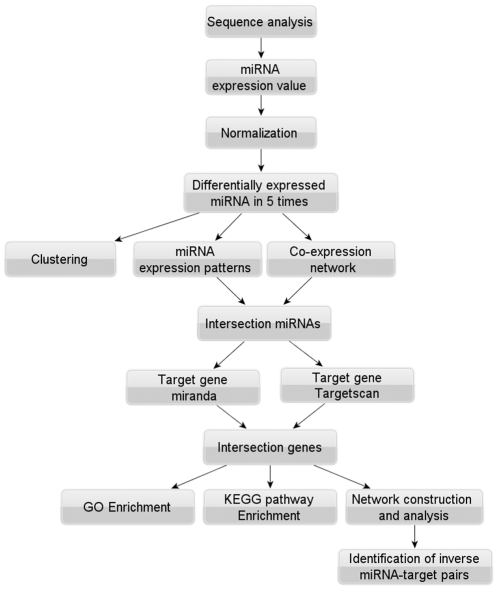
The overall procedure of combinatorial approach to annotate known miRNA and identify their targets.

In this work, we have designed an integrative strategy with bioinformatic analysis to identify miRNAs in the DRG and the proximal stump response to resection of the sciatic nerve in a rat model by deep sequencing (Figure 1). We found 201 and 225 known miRNAs with significant expression variance in the DRG and the proximal stump after nerve injury, respectively. More importantly, our study screened out some key miRNAs, through bioinformatics analysis, that may be involved in many aspects of nerve repair, and provided an opportunity to decipher what molecules they target to regulate nerve regeregation by integration of predicted targets with differentially expressed mRNAs, suggesting that miRNA play important roles in expression regulation responsible for a series of concerted actions of DRG neurons and SCs for formation of new nerve tissue.

## Results

### Construction of Ten Different Small RNA Libraries by Solexa Sequencing

**Figure 2:**
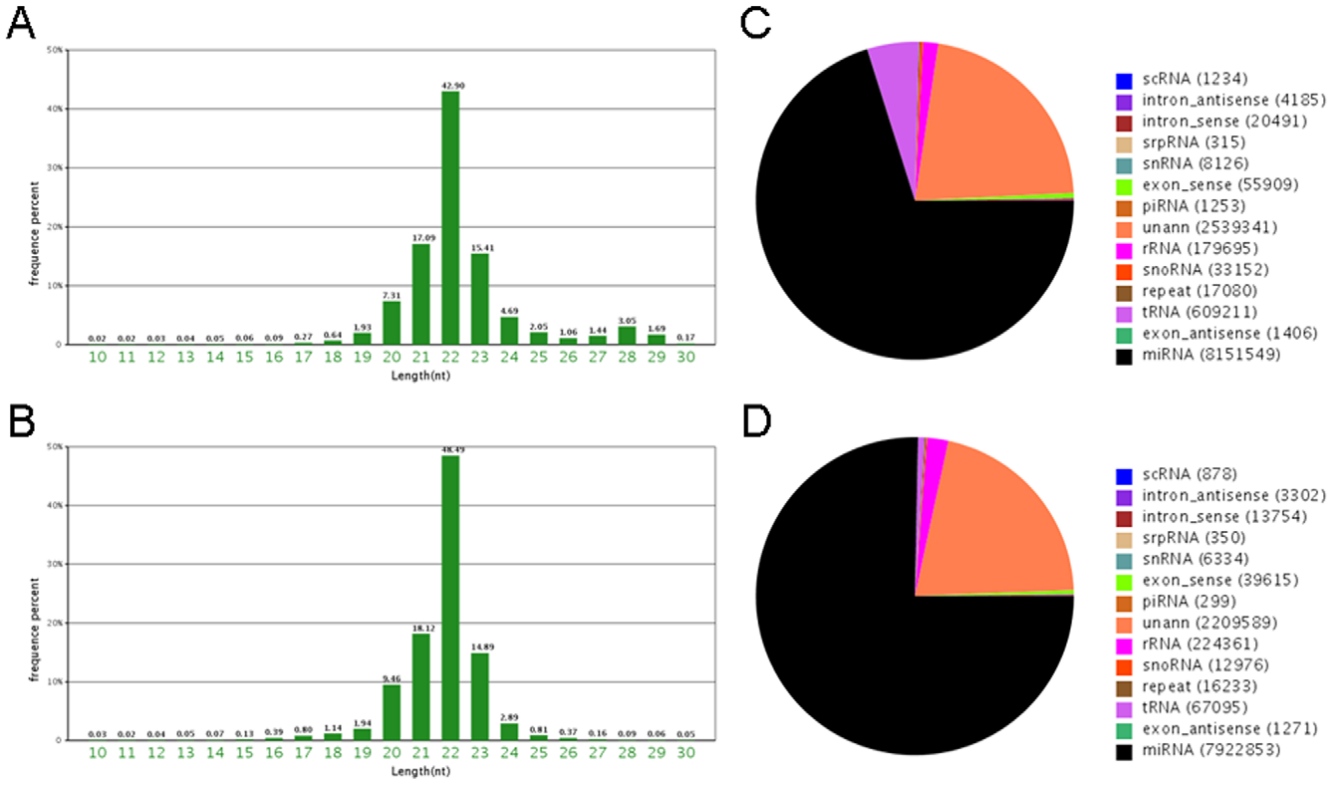
Size and frequency distribution of the sequencing reads, and calssification of small RNAs. A. Length distribution of the non-redundant sequencing reads from normal DRG. B. Length distribution of the non-redundant sequencing reads from normal SN. C. Classification of the sequenced small RNA tags from normal DRG. D. Classification of the sequenced small RNA tags from normal SN.

In order to identify the miRNAs in DRG tissues (DRG) and the proximal stumps of the sciatic nerve (SN) at different time points following rat sciatic nerve transection, ten different small RNA libraries from the model were sequenced using Solexa technology. After removing the reads of low quality and masking adaptor sequences, total reads of 10 to 30 nucleotides in length were obtained from ten samples (Table S1). A dataset of about 22 million reads (11900408 and 10790160 reads isolated from DRG and SN tissues at 0 d, respectively), ranging from 18 to 30 nt was obtained after trimming the adapter sequences. From the size distribution of total reads, we found the length distribution peaked at 22 nucleotides and almost half of these clean reads (42.90% and 48.49%, respectively) were 22 nucleotides in length, consistent with the common size of miRNA (Figure 2A and 2B). We then further excluded these small RNAs that matched with known rRNAs, tRNAs, small nuclear RNAs (snRNAs) and small nucleolar RNAs (snoRNAs) deposited in the Rfam database (ftp://selab.janelia.org/pub/Rfam) and NCBI GenBank (http://www.ncbi.nlm.nih.gov/GenBank). We also masked the repeat sequences using Exonhunter (http://lifecenter.sgst.cn/schistosoma/en/schistosomaCnIndexPage.do) and RepeatMasker programs (http://www.repeatmasker.org), and those mapped to protein coding exons. After removing the above small RNAs, a total of 8151549 and 7922853 tags from DRGs and SNs were further analyzed for the identification of rat miRNAs (Table S1). This result implies an enrichment of miRNA in the small RNA library of DRG tissues (Figure 2C) and SN tissues (Figure 2D).

### Category Analysis of Specific MiRNAs

**Figure 3:**
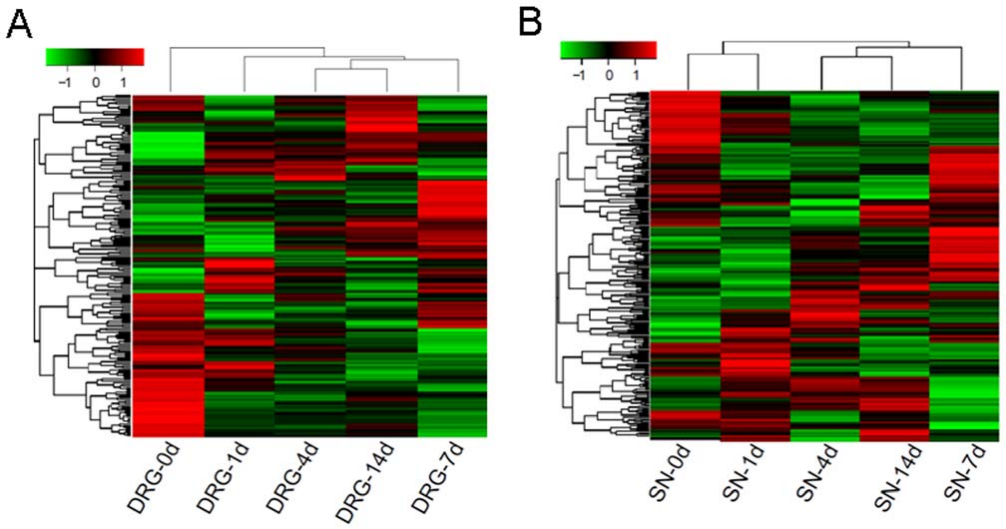
The heatmap for the miRNA with significant expression variance. A. Heatmap and cluster dendrogram of differentially expressed 201 miRNAs from DRG with or without injury. B. Heatmap and cluster dendrogram of differentially expressed 225 miRNAs from SN with or without injury. The color scale shown on the top illustrates the relative expression level of the indicated miRNA across all samples: red denotes expression >0 and green denotes expression <0.

Based on pervious criteria, a total of 330 known rat miRNA genes were identified. The number of differentially expressed miRNAs with different false detection rate (FDR) was counted in Table S2 and Table S3. In detail, there are 201 and 225 miRNAs with significant expression variance identified in DRG and SN tissues with qFDR <0.05, respectively. We then conducted the clustering for each tissue by hierarchical cluster, and the results of hierarchical cluster were shown by heatmap. The miRNA transcriptome of these two groups was significantly different between 4 d, 7 d, 14 d and 0 d, 1 d after sciatic nerve injury in either DRG tissues (Figure 3A) or SN tissues (Figure 3B).

**Figure 4:**
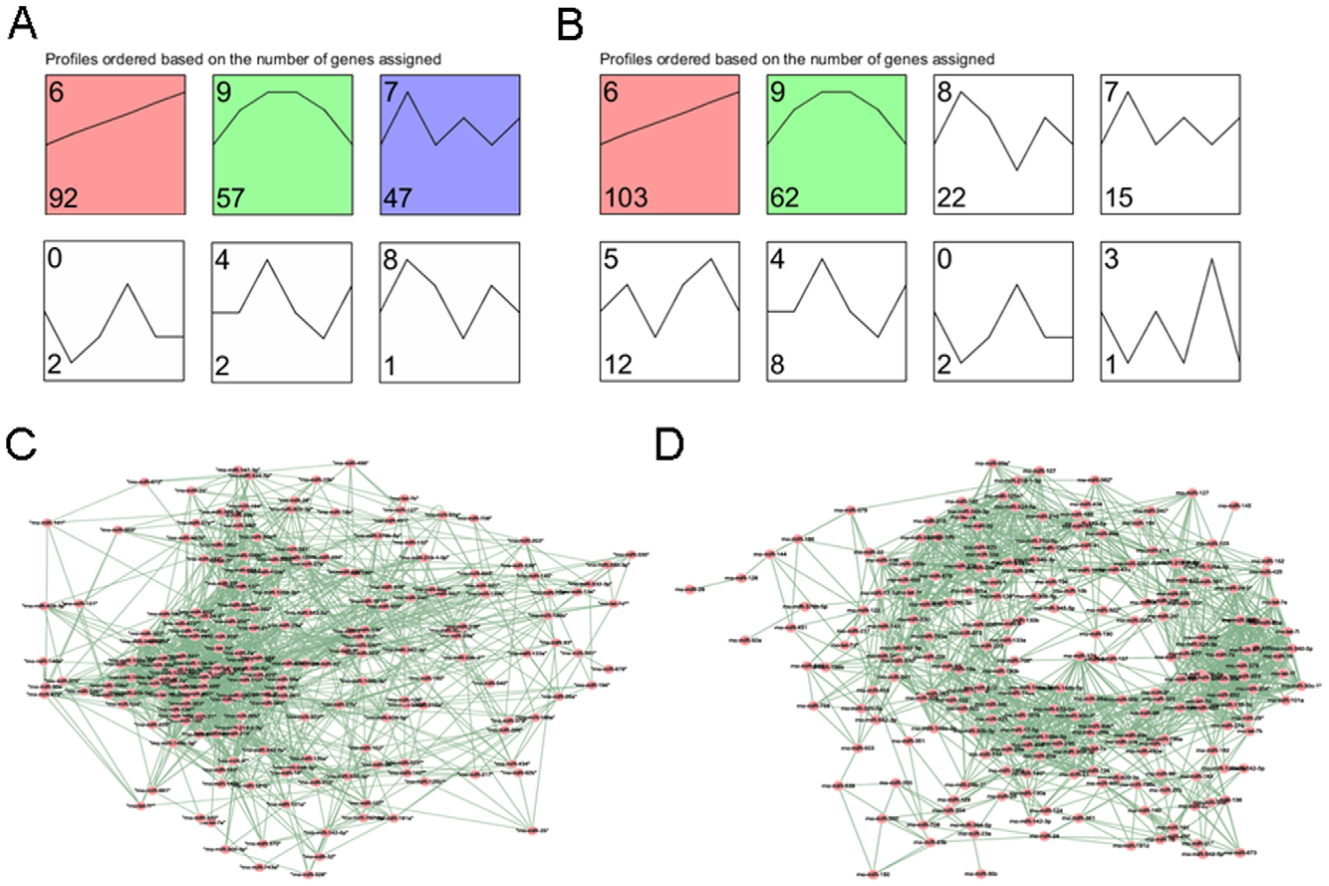
The expression pattern for differentially expression miRNAs and co-expression network. A. The expression pattern for 201 differentially expression miRNAs from DRG. B. The expression pattern for 225 differentially expression miRNAs from SN. C. Co-expression network of 201 miRNAs from DRG. D. Co-expression network of 225 miRNAs from SN.

In order to screen out some key miRNAs, we performed expression pattern and co-expressed network for these two groups of miRNA. Firstly, 3 (profile 6, 9, 7) and 2 (profile 6, 9) significant expression patterns were found for DRG tissues (Figure 4A) and SN tissues (Figure 4B). The pattern of profile 6 was up-regulated along the time series, but the pattern of profile 9 was first up- then down-regulated, compared to that for the control group, throughout a period of 14 d. There were 92, 103 miRNAs in profile 6 and 57, 62 miRNAs in profile 9 in DRG and SN tissues, respectively. Secondly, we got 200 and 224 co-expressed miRNAs (Figure 4C and 4D), that had correlation (|cor| ≥0.9) with at least one other miRNA. Finally, the intersection of profiles 6, 9 and co-expressed miRNAs, which have at least 20 edges, were selected. Specifically, D6 (DRG tissue, profile 6) included 19 miRNAs, D9 (DRG tissue, profile 9) 4 miRNAs, S6 (SN tissue, profile 6) 36 miRNAs and S9 (SN tissue, profile 9) 19 miRNAs.

### Function Annotation for Target Genes of the miRNA

To further understand the biological function of these miRNAs, the target genes of the above categories of miRNA were predicted by Targetscan and miRanda. We got 690, 135, 775 and 408 intersected targets for conserved miRNAs of four categories (Table S4, Table S5, Table S6, Table S7), respectively. In order to get credible biological functions, we conducted the GO (Table 1) and KEGG pathway (Table S8) enrichment analyses just for the intersected genes. Table 1 lists the GO terms that have the highest enriched score and most significant *P* value. The most significant GO functions (cell morphogenesis involved in differentiation; neuron differentiation; cell adhesion and negative regulation of cellular process) were significantly enriched by the targets of 19 miRNAs in D6. The most significant GO functions (regulation of localization; vesicle-mediated transport and anti-apoptosis) were significantly enriched by the targets of 4 miRNAs in D9. In summary, the most significant GO functions of miRNAs in D6 and D9 are all neuron-regeneration related, including anti-apoptosis, vesicle-mediated transport and neuron differentiation. Moreover, the most significant GO functions (establishment of localization and neurogenesis) were significantly enriched by the targets of 36 miRNAs in S6. The most significant GO functions (homophilic cell adhesion; negative regulation of programmed cell death; cation transport and synaptic transmission) were significantly enriched by the targets of 19 miRNAs in S9. The most significant GO functions of miRNAs in S6 and S9 are SC survival and function related, including negative regulation of programmed cell death, homophilic cell adhesion and neurogenesis. Functional classification by KEGG pathway database also shows that the most significant pathway enriched by the targets of 19 miRNAs and 4 miRNAs in D6 and D9 are highly associated with axon regeneration, including the neurotrophin signaling pathway, MAPK signaling pathway and adherens junction, whereas the most significant pathway enriched by the targets of 36 miRNAs and 19 miRNAs in S6 and S9 are highly associated with SC dedifferentiation, proliferation, migration and re-differentiation, including the TGF-beta signaling pathway, adherens junction, cell cycle and MAPK signaling pathway.

**Table 1:** The most significant GO functions for miRNA targets.

GO ID	GO terms	*P* value	qFDR
D6			
GO:0007275	multicellular organismal development	1.35E-11	3.64E-08
GO:0022008	neurogenesis	9.68E-11	1.31E-07
GO:0051234	establishment of localization	2.06E-09	1.85E-06
GO:0000904	cell morphogenesis involved in differentiation	1.34E-08	9.01E-06
GO:0030182	neuron differentiation	5.80E-08	2.64E-05
GO:0065008	regulation of biological quality	6.85E-08	2.64E-05
GO:0032990	cell part morphogenesis	6.94E-08	2.64E-05
GO:0007155	cell adhesion	7.82E-08	2.64E-05
GO:0007156	homophilic cell adhesion	1.56E-07	4.67E-05
GO:0048523	negative regulation of cellular process	3.49E-07	9.41E-05
D9			
GO:0016044	membrane organization	9.68E-06	0.015
GO:0032879	regulation of localization	3.20E-05	0.023
GO:0016192	vesicle-mediated transport	4.55E-05	0.023
GO:0006916	anti-apoptosis	7.10E-05	0.024
GO:0051674	localization of cell	7.97E-05	0.024
S6			
GO:0051234	establishment of localization	7.67E-14	2.11E-10
GO:0051254	positive regulation of RNA metabolic process	1.45E-10	1.99E-07
GO:0045944	positive regulation of transcription from RNA polymerase II promoter	2.26E-10	2.07E-07
GO:0045941	positive regulation of transcription	5.44E-10	3.74E-07
GO:0022008	neurogenesis	7.58E-10	4.17E-07
GO:0032774	RNA biosynthetic process	2.10E-09	9.64E-07
GO:0009891	positive regulation of biosynthetic process	7.79E-09	2.83E-06
GO:0031325	positive regulation of cellular metabolic process	8.23E-09	2.83E-06
GO:0019222	regulation of metabolic process	9.64E-09	2.95E-06
GO:0010468	regulation of gene expression	1.37E-08	3.78E-06
S9			
GO:0007156	homophilic cell adhesion	1.79E-08	4.03E-05
GO:0008016	regulation of heart contraction	2.60E-07	0.0003
GO:0043069	negative regulation of programmed cell death	4.42E-07	0.0003
GO:0003015	heart process	6.60E-07	0.0003
GO:0008219	cell death	6.78E-07	0.0003
GO:0006812	cation transport	1.28E-06	0.0005
GO:0007268	synaptic transmission	7.26E-06	0.002
GO:0007611	learning or memory	1.82E-05	0.005
GO:0042592	homeostatic process	2.01E-05	0.005
GO:0032502	developmental process	2.14E-05	0.005

**Figure 5:**
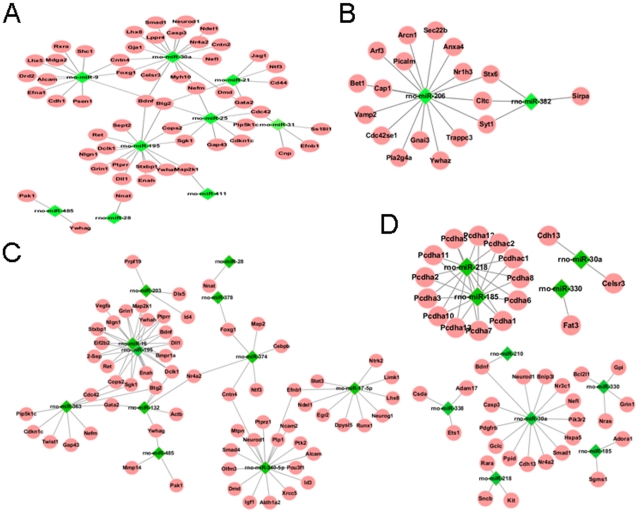
The subnetwork for target genes mapped in particular GO terms and the miRNAs. A. The network of target genes in neuron differentiation and miRNAs of D6. B. The network of target genes in vesicle-mediated transport and miRNAs of D9. C. The network of target genes in neurogenesis and miRNAs of S6. D. The network of target genes in homophilic cell adhesion and negative regulation of programmed cell death and miRNAs of S9.

We additionally constructed networks for target genes that mapped the particular GO terms and miRNAs. The network of target genes in neuron differentiation of D6 are shown in Figure 5A, the vesicle-mediated transport of D9 in Figure 5B, the neurogenesis of S6 in Figure 5C and the homophilic cell adhesion, negative regulation of programmed cell death of S9 in Figure 5D. These results indicated that miRNA play important roles in nerve regeneration by down-regulating these proteins in different expression pattern profiles.

### Selection of Inverse MiRNA-Target Pairs

miRNAs are generally thought to negatively regulate the expression of their targets by mRNA degradation or by repressing translation. If a given miRNA is up-regulated, the expression of its targets is expected to be down-regulated. cDNA expression profiling data were consequently integrated to identify miRNA targets [18], [19]. We then combined the intersected targets for every category with differentially expressed mRNAs and selected only the miRNA-target pairs that were expressed in DRG and SN tissues after nerve injury and inversely correlated.

We identified 22 miRNA-target pairs that are potentially implicated in nerve regeneration (Table 2). Specifically, miR-195 potentially regulates vesicle-associated membrane protein 1 (VAMP1), miR-30a targets actinin, alpha 1 (ACTN1), miR-21 targets paired-like homeodomain 2 (PITX2) in D6; miR-132 potentially regulates solute carrier family 2, member 1 (SLC2A1), nuclear receptor subfamily 4, group A, member 2 (NR4A2) and Cdc42 guanine nucleotide exchange factor 9 (ARHGEF9), miR-203 targets calcium binding protein 7 (CABP7), miR-17-5p targets early growth response 2 (EGR2) in S6; miR-330 potentially regulates CD247, nerve growth factor receptor (NGFR) and FAT tumor suppressor homolog 3 (FAT3), miR-338 targets ADAM metallopeptidase domain 17 (ADAM17), miR-218 targets src kinase associated phosphoprotein 1 (SKAP1), miR-185 targets calcium channel, voltage-dependent, N type, alpha 1B subunit (CACNA1B) in S9.

**Table 2:** Selected inverse miRNA-target relation identified.

miRNA	Target gene	Pearson's r	*P* value
D6			
rno-mir-25	PER2	-0.912	0.031
rno-mir-195	VAMP1	-0.885	0.046
rno-mir-30a	ACTN1	-0.837	0.077
rno-mir-21	PITX2	-0.822	0.087
S6			
rno-mir-132	SLC2A1	-0.908	0.033
rno-mir-17-5p	DDHD1	-0.908	0.033
rno-mir-17-5p	CMTM6	-0.855	0.065
rno-mir-132	NR4A2	-0.855	0.065
rno-mir-132	ARHGEF9	-0.855	0.065
rno-mir-203	CABP7	-0.844	0.072
rno-mir-17-5p	EGR2	-0.814	0.094
S9			
rno-mir-330	CD247	-0.962	0.009
rno-mir-338	CIITA	-0.935	0.020
rno-mir-338	ADAM17	-0.932	0.021
rno-mir-218	DBNDD1	-0.920	0.027
rno-mir-218	SKAP1	-0.919	0.027
rno-mir-338	UPK1B	-0.909	0.032
rno-mir-330	NGFR	-0.900	0.037
rno-mir-330	FAT3	-0.865	0.058
rno-mir-330	DTNB	-0.828	0.083
rno-mir-185	CACNA1B	-0.809	0.097
rno-mir-218	TMEM178	-0.809	0.097

## Discussion

Crush injury or axotomy to the peripheral nerves results in a sequence of molecular and cellular responses, which may reactivate the intrinsic growth capacity of neurons and orchestrate a regenerative process. Moreover, the proximal stump could maintain the structural and functional integrity except a retrograde degeneration in a short segment [20]. The search for the master regulators and related target genes responsible for these cellular reactions and underlying molecular mechanisms is consecutively one of the most important areas in nerve regeneration research. The survival and axon growth of DRG neurons and the regulative capability of SCs have been the subject of extensive research for years [21]. However, the underlying molecular mechanisms that elaborate the concerted action of DRG neurons and SCs through system-level analysis have attracted no attention. Decoding of the variations of molecular regulation in DRG and the proximal nerve segments following sciatic nerve injury not only contribute to the promotion of peripheral nerve regeneration, but may also provide treatment strategies for demyelinating neuropathies.

To understand the issue, large-scale analysis of gene expression pattern is required. The microarray technique offers the possibility to study the regulation of thousands of genes simultaneously, achieving a comprehensive overview of gene activity after injury and during regeneration. Such studies have previously been performed on DRG and sciatic nerve following nerve injury [22]–[24], while the master regulators responsible for the dynamic change of gene expression remain largely unclear. We have more recently focused on miRNA since a single miRNA has the potential to target hundreds of distinct mRNA molecules and one mRNA molecule can be regulated by multiple miRNAs [25], which means miRNAs are attractive candidates as regulators of multiple pathways. It has, however, become increasingly clear that not all miRNAs are equally important. Particular miRNAs emerge as principal regulators that control major cell functions in various physiological and pathophysiological settings [26]. Hence, the investigation of miRNA alteration in DRG and SN tissues following sciatic nerve resection will be crucial in acquiring knowledge about the basic mechanisms of nerve regeneration and in identifying these particular miRNAs as the triggers and regulators through system-level analysis. In the currently available miRNA test, miRNA array analyses exhibit low sensitivity and specificity. Deep sequencing was recently introduced and significantly increased the accuracy of the sequence [17].

**Figure 6:**
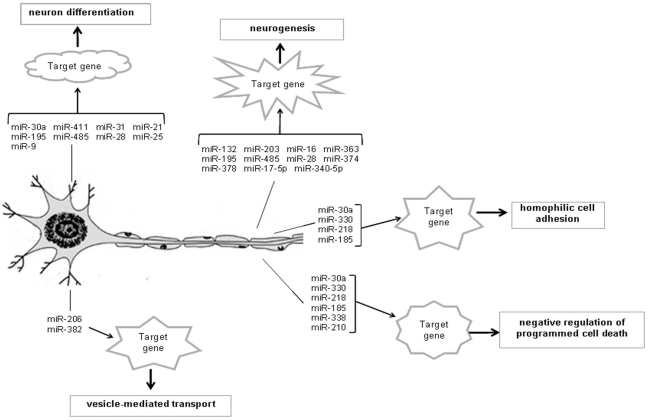
Schematic representation of the role of miRNAs during nerve regeneration.

The aim of this study is to systematically characterize miRNAs in DRG tissues and the stumps of the nerve following sciatic nerve resection using deep sequencing. Our results demonstrate that miRNAs are abundant in these tissues and show significant changes after axotomy to the peripheral nerves. We screened out some specific miRNAs, obtained the intersection genes through target analysis software, and performed GO and KEGG enrichment analyses for the targets. Interesting, the most significant GO functions for miRNA targets and the most significant pathway for miRNA targets are highly associated with nerve regeneration. Based on the results, the overall effects of miRNA in mediating the injury-induced regeneration of sciatic nerve is summarized in Figure 6. We proposed a unique mechanism by which injury causes deregulation of these miRNAs, which regulate the action of DRG neuron and SC from multi-angles, leading to formation of new nerve tissue. Because the regulatory mechanisms through miRNA generally involve a network, there are several important miRNA and miRNA-target pairs that were obtained from the system-level analysis for each subnetwork.

It is known that the intrinsic growth potential of neurons is determined by the activity of specific molecular sets, which are interconnected in a vastly complicated network [21], [27]. At the same time, the expression and function of these molecules are dynamically regulated by multiple mechanisms, which adjust the actual growth properties of each neuron population at different ontogenetic stages or in injury conditions. Interestingly, neuron differentiation and vesicle-mediated transport are the most representative GO functions enriched by the targets of miRNAs in D6 and D9, consistent with the notion that regeneration is at least a partial re-capitulation of molecular and cellular mechanisms occurring during development [28].

Indeed, the molecules that possess neuroprotective properties and mediate repair of damaged nerve were screened out in different investigations. Brain-derived neurotrophic factor (BDNF) plays a prominent role in promoting axonal growth of motor and sensory neurons [29], [30], and endogenous BDNF is required for peripheral nerve regeneration and remyelination after nerve injury; however, high doses of BDNF inhibited regeneration in acute nerve injury [31]. Specially, miR-195 and miR-30a could contribute to the regulation of BDNF expression in human prefrontal cortex [32]. In our study, miR-195 and miR-30a were promptly elevated at 1 d after sciatic nerve injury, and then leveled off with significantly higher values, compared to that for the control group, throughout a period of 14 d. It is therefore intriguing to speculate that miRNAs, including miR-195 and miR-30a, might participate in orchestrating and fine-tuning BDNF signaling in nerve regeneration. VAMP1, the target of miR-195, was involved in synaptic vesicle recycling and exocytosis in neurons [33]. Meanwhile, differential regulation of VAMPs, which were transiently altered from 3 to 21 days after axotomy of facial nerve, may reflect distinct roles in nerve regeneration. Interestingly, ACTN1, the target of miR-30a, is an actin-binding protein with multiple roles in different cell types. In non-muscle cells, the cytoskeletal isoform was found along microfilament bundles and adherens-type junctions, where it is involved in binding actin to the membrane [34].

MiR-21 was also in the subnetwork of neuron differentiation. The expression of miR-21 is significantly up-regulated in the hippocampus after traumatic brain injury in rodents, with expression level peaking by 3 days post-injury and returning to near-sham level by 15 days post-injury [35]. Moreover, miR-21 expression was also significantly up-regulated following traumatic spinal cord injury in another recent report [11], the results of which are similar to our study. PITX2, the target of miR-21 listed in Table 2, was demonstrated to regulate regionally specific terminal neuronal differentiation in the developing ventrolateral thalamus and midbrain and to be involved in the control of retina regeneration [36], [37]. PTEN was shown to be a direct target of miR-21 [38]. Interestingly, PTEN inhibition enhances the regenerative ability of adult corticospinal neurons and facilitates intrinsic regenerative outgrowth of adult peripheral axons [39], [40]. Modulation of the PTEN/mTOR pathway can promote axon regeneration in the adult CNS [41]. It is, therefore, intriguing to speculate that miRNAs, including miR-195, miR-30a and miR-21 (in D6), may participate in a molecular network involving multiple reciprocal nodes that together orchestrate and fine-tune diverse targets and signaling in axon outgrowth.

Neuronal response to injury, as well as neuronal development, depend on both intrinsic programs of gene expression and on extrinsic cues from the surrounding environment. In general, the proximal stump (mainly SCs) is endowed with some autonomous regulative capabilities in the maintenance of neuronal survival, neuritic growth and myelination of the regenerative axon [42]. The failure support of SCs always resulted in the axonal degeneration observed in human neurological diseases. Interestingly, neurogenesis is the most representative GO function enriched by the targets of miRNAs in S6; both homophilic cell adhesion and negative regulation of programmed cell death are the most significant GO functions enriched by the targets of miRNAs in S9.

SCs activate, lose their myelin sheaths and dedifferentiate after nerve injury. Activated SCs can proliferate, remove degenerated axonal and myelin debris and migrate to form SC columns that also produce various trophic factors and adhesion molecules and offer a constructive environment for axon regeneration [43]. MiR-330 is reported to induce apoptosis in prostate cancer cells through E2F1-mediated suppression of Akt phosphorylation [44], and in our subnetwork NGFR (also known as P^75^NTR) is the target of miR-330. Proliferation of SC reaches a peak within 7 d post-injury split, and then undergoes apoptosis under the roles of NGF and P^75^NTR [45], which contributes to sustain the dynamic balance in the number of SCs. In the cell adhesion subnetwork, CD247, the target of miR-330, plays an important role in coupling antigen recognition to several intracellular signal-transduction pathways [46]. Especially, SKAP1, the target of miR-218, plays a critical role in inside-out signaling by coupling T-cell antigen receptor stimulation to the activation of integrins, which are transmembrane adhesion molecules that mediate cell-cell or cell-extracellular matrix adhesion [47].

Meanwhile, by accepting specific molecular signals from the SCs, neurons extend axons and dendrites using glia as “guide posts” along appropriate SC processes and to form proper synaptic connections [6]. In the general neurogenesis subnetwork, the nuclear orphan receptor NR4A2, the target of miR-132, are induced after excitotoxic and oxidative stress, up-regulate neuroprotective genes, and increase neuronal survival. Moreover, NR4A proteins are induced by cAMP response element binding protein (CREB) when exposed to stressful insults and function as mediators of CREB-induced neuronal survival [48]. In addition, NR4A2 controls the differentiation of selective dopaminergic nuclei in the zebrafish brain [49]. SLC2A1, another target of miR-132, is expressed in the plasma membrane and cytoplasm of myelinating SCs around the nodes of Ranvier and in the Schmidt-Lanterman incisures, and is involved in the transport of glucose into the metabolically active region of peripheral axons [50]. In mammals, it has been recently revealed that miR-132 was able to modulate dendritic morphology via suppression of specific targets [51], and miR-132 increased dendritic protrusion width and increased miniature excitatory postsynaptic current amplitude [52]. Consequently, SCs may alter miRNA expression, including miR-330, miR-218 (in S9) and miR-132 (in S6), to modulate two-way communication between neurons and SCs to maintain structural integrity and functional recovery after axotomy to the peripheral nerves.

In addition, the hierarchical cluster analysis of the control and regenerating samples showed tight clustering (Figure 3). In particular, the miRNA transcriptome in both DRG and SN tissues show an extraordinary resemblance between 4 d and 14 d after nerve injury, and the miRNA transcriptome at 7 d is also close to the 4 d and 14 d, indicating that nerve regeneration seems to reach its peak at 7 d after resection of the sciatic nerve in a rat model. Outgrowth studies are necessarily limited to the interval of 4–7 days [3], [4], [39]. Our study may provide an opportunity to divide stages of nerve regeneration from the level of small RNA molecules.

To our knowledge, this is the first study of dynamic changes in miRNA expression in DRG and the nerve stump in the interval of 1 d –14 d after transection of the sciatic nerve by deep sequencing. The bioinformatics analysis indicated that the specific miRNAs and miRNA-target pairs were potentially involved in the key processes of mobilizing an intrinsic capacity for neurite outgrowth, modulating SC phenotype, coordinating SC-neuron communication and promoting nerve repair. Our results provide a new insight into the molecular basis of nerve repair through system-level analysis, which suggests that miRNA may critically contribute to the regulating of nerve regeneration and may, therefore, be potential targets for therapeutic intervention following nerve injury.

## Materials and Methods

### Animal Surgery and Tissue Preparation

Thirty adult, male Sprague-Dawley (SD) rats (180–220 g, supplied by the Experimental Animal Center of Nantong University) were randomly divided into five groups of six rats each. Each animal was anaesthetized by an intraperitoneal injection of complex narcotics (85 mg/kg trichloroac etaldehyde monohydrate, 42 mg/kg magnesium sulfate, 17 mg/kg sodium pentobarbital), and the sciatic nerve was exposed and lifted through an incision on the lateral aspect of the mid-thigh of the left hind limb. A 1 cm long segment of sciatic nerve was then resected at the site just proximal to the division of tibial and common peroneal nerves, and the incision sites were then closed. To minimize discomfort and possible painful mechanical stimulation, the rats were housed in large cages with sawdust bedding after surgery. L4-6 DRG tissues and SN tissues (0.5 cm) were collected at different time points after injury, respectively. All the experimental procedures involving animals were conducted in accordance with Institutional Animal Care guidelines and ethically approved by the Administration Committee of Experimental Animals, Jiangsu Province, China (Approval ID: SYXK(SU)2007-0021).

### RNA Extraction, Construction of Small RNA Libraries, and Sequencing

Total RNA was extracted using the mirVana™ miRNA Isolation Kit (Ambion, Austin, TX) according to the manufacturer's instructions. The quality of the purified RNA was assessed using a BioAnalyzer 2100 (Agilent Technology, Santa Clara, CA). The purified RNA was quantified by determining the absorbance at 260 nm using a Nanodrop ND-1000 spectrophotometer (Infinigen Biotechnology Inc., City of Industry, CA). RNA samples were stored at −80°C. A total of ten samples of DRG and SN tissues were collected at 0 d, 1 d, 4 d, 7 d and 14 d after injury, respectively, and were sequenced with Solexa/Illumina platform by BGI (Beijing Genome Institute at Shenzhen).

For small RNA library construction and deep sequencing, the 10–30 nt size range of RNA was enriched by polyacrylamide gel electrophoresis and then 20 µg of the purified small RNA from each sample was subject to DNA sequencing with an Illumina Genome Analyzer (Illumina, San Diego, CA) according to the manufacturer's instructions. In brief, proprietary adapters were then ligated to the 5′ and 3′ termini of these small RNAs, of which the ligated small RNAs were then used as templates for cDNA synthesis. The cDNA was amplified with 18 PCR cycles to produce libraries that were sequenced using Solexa's proprietary sequencing-by-synthesis method. The image files generated by the sequencer were then processed to produce digital-quality data. After masking of adaptor sequences and removal of contaminated reads, we got the clean reads of full-length small RNA sequences for further analysis. To control the quality of these sequencing data, we calculated the average quality score of sites and reads of each sample.

### Search for Specific Profile of miRNAs

Our strategy was as follows: investigating all small RNAs expressed in ten samples via Solexa, computationally scanning the rat genome for candidate hairpin miRNA genes corresponding to Solexa reads using MIREAP and identifying conserved miRNA genes using miRAlign. We then identified the differentially expressed miRNAs having large variant expression among 5 times by Chi-Square Test. The *P* value here was also adjusted by FDR. In addition, the method used for normalizing expression data of miRNA was quantile normalization.

Using the miRNAs with significant expression variance, we conducted the following analysis: 1) Hierarchical clustering with the expression of these miRNAs. We calculated Z-score from the expression of miRNAs. The euclidean distance measure was used to compute the distance (dissimilarity) in both ways (miRNAs and times). 2) MiRNA expression patterns clustering. The Short Time-series Expression Miner (STEM: http://www.cs.cmu.edu/~jernst/stem/) was used to find the expression patterns. 3) Co-expression analysis between miRNAs. We calculated the Pearson correlation coefficient between miRNAs and selected correlated miRNA with absolute correlation coefficient ≥0.9, and built the network for these miRNAs. We then searched for the intersection of co-expression miRNAs with edge ≥20 and expression pattern profile 6 or profile 9 for DRG and SN tissues.

### Annotation of miRNA Targets

Two types of miRNA target prediction software, TargetScan (http://www.targetscan.org), and miRanda (http://www.microrna.org/microrna/home.do), were used to predict the target genes of these specifically expressed miRNAs. In order to get more credible biological functions, we used gene ontology (GO) and Kyoto Encyclopedia of Genes and Genomes (KEGG) enrichment analyses for the intersected targets for conserved miRNAs. R package was used for the analysis and *P* value was adjusted by FDR. Afterwards, the regulated network for conserved miRNAs and the targets was constructed, and the networks were built for the target genes in the particular GO terms and the miRNAs that regulated these genes.

### Integration of miRNAs with Serial Analysis of Gene Expression Data

In our analysis, DRG and SCs at the same time points were sampled for mRNA expression profile scanning by microarray after segmentation of the peripheral nerve. All data is MIAME compliant and the raw data has been deposited in a MIAME compliant database GEO, as detailed on the MGED Society website: http://www.mged.org/Workgroups/MIAME/miame.html. Two groups of data were compared, the significance (*P* value) and FDR were calculated using the adjusted F-test with the Random Variation Model (RVM), and the differentially expressed genes in these serial time points were obtained via screening. We then integrated our specific miRNAs and serial analysis of gene expression data and selected only the miRNA-target pairs that were inversely correlated. We calculated the Pearson correlation coefficient and *P* value between miRNAs and mRNAs. The mRNAs were thought to be down-regulated by the miRNAs when the *P* value <0.1.

## Supporting Information

Table S1 **Sequence analysis of ten different small RNA libraries.**
(XLS)

Table S2 **Differentially expressed miRNA in DRG.**
(XLS)

Table S3 **Differentially expressed miRNA in SN.**
(XLS)

Table S4 **Intersected targets for conserved miRNA of D6.**
(XLS)

Table S5 **Intersected targets for conserved miRNA of D9.**
(XLS)

Table S6 **Intersected targets for conserved miRNA of S6.**
(XLS)

Table S7 **Intersected targets for conserved miRNA of S9.**
(XLS)

Table S8 **The most significant pathway for miRNA targets.**
(XLS)
